# Cutaneous Mastocytosis in Childhood—Update from the Literature

**DOI:** 10.3390/jcm10071474

**Published:** 2021-04-02

**Authors:** Florica Sandru, Răzvan-Cosmin Petca, Monica Costescu, Mihai Cristian Dumitrașcu, Adelina Popa, Aida Petca, Raluca-Gabriela Miulescu

**Affiliations:** 1 “Carol Davila” University of Medicine and Pharmacy, 030167 Bucharest, Romania; florysandru@yahoo.com (F.S.); drpetca@gmail.com (R.-C.P.); monica.costescu@yahoo.com (M.C.); miulescuraluca@yahoo.com (R.-G.M.); 2 Department of Dermatology, Elias University Emergency Hospital, 0611461 Bucharest, Romania; adelinaade541@yahoo.com; 3 Department of Urology, “Prof. Dr. Theodor Burghele” Clinical Hospital, 061344 Bucharest, Romania; 4 Department of Dermatology, “Dr. Victor Babes” Clinical Hospital of Infectious and Tropical Diseases, 030303 Bucharest, Romania; 5 Department of Obstetrics & Gynecology, University Emergency Hospital, 050098 Bucharest, Romania; 6 Department of Obstetrics & Gynecology, Elias University Emergency Hospital, 0611461 Bucharest, Romania; 7 Department of Dermatology, Vălenii de Munte Hospital, 106400 Prahova, Romania

**Keywords:** mastocytosis, cutaneous mastocytosis, systemic mastocytosis, children, antihistamines, PUVA, cromolyn sodium, urticaria pigmentosa

## Abstract

Mastocytosis (M) represents a systemic pathology characterized by increased accumulation and clonal proliferation of mast cells in the skin and/or different organs. Broadly, M is classified into two categories: Cutaneous mastocytosis (CM) and systemic mastocytosis (SM). In children, CM is the most frequent form. Unfortunately, pathogenesis is still unclear. It is thought that genetic factors are involved, but further studies are necessary. As for features of CM, the lesions differ in clinical forms. The most important fact is evaluating a pediatric patient with CM. It must comprise laboratory exams (with baseline dosing of total serum tryptase), a skin biopsy (with a pathological exam and, if the diagnosis is unclear, immunohistochemical tests), and a complete clinical evaluation. It is also defining to distinguish between CM and other diseases with cutaneous involvement. As for the management of CM in children, the first intervention implies eliminating trigger factors. The available cures are oral H1 and/or H2 antihistamines, oral cromolyn sodium, oral methoxypsoralen therapy with long-wave psoralen plus ultraviolet A radiation, potent dermatocorticoid, and calcineurin inhibitors. In children, the prognosis of CM is excellent, especially if the disease’s onset is in the first or second years of life.

## 1. Introduction

Mastocytosis (M) represents a systemic pathology characterized by increased accumulation and clonal proliferation of mast cells in the skin and/or different organs. Broadly, M is classified into two categories: Cutaneous mastocytosis (CM) and systemic mastocytosis (SM) (affecting extracutaneous tissues—spleen, liver, bone marrow, and lymph nodes) [1].

Nettleship and Tay first mentioned the term mast cell pathology in 1869 [2]. Later, in 1878, Sangester named this disease urticaria pigmentosa (UP) [3]. The real discovery was made by Ellis in 1949 when he found mast cells to infiltrate the skin and extracutaneous organs such as the liver, spleen, bone marrow, and lymph nodes [4]. 

### Classification of Mastocytosis

The latest classification was issued by the World Health Organization in 2019 and included:

- Cutaneous mastocytosis—with the following forms: Diffuse cutaneous mastocytosis, maculopapular cutaneous mastocytosis, urticaria pigmentosa (UP), and solitary skin mastocytoma;
- Systemic mastocytosis, associated through a clonal mechanism with hematological neoplasm;
- Indolent systemic mastocytosis: It does not associate with hematological neoplasm;
- Mast cell sarcoma: Localized destructive growth pattern; does not associate with systemic mastocytosis;
- Systemic mastocytosis—the aggressive form; does not associate with mast cell leukemia;
- Smoldering systemic mastocytosis;
- Mast cell leukemia: Diffuse and dense infiltration, atypical, immature mast cells are evidenced at bone marrow biopsy; bone marrow aspirate: >20% mast cells; mast cells >10% of peripheral blood white cells (classic form) [5,6,7].

## 2. Main Features of CM in Childhood

### 2.1. Epidemiology of CM in Childhood

CM was first described by Sezary and Chauvillon in 1936 [8]. It is most common in children, representing 90% of all cases in this age group [9]. Maculopapular cutaneous mastocytosis represents the cutaneous form. This has the highest prevalence and includes UP and other nodular-plaque forms. CM presents a bimodal distribution: UP manifests itself in children aged one year and is diagnosed before two years old. On the other hand, young children over the age of 15 develop mastocytomas [10].

### 2.2. Pathophysiology of CM in Childhood

The pathogenesis of CM in children is still unclear, in contrast to the mechanisms found in adult patients with this systemic form of the disease. It is thought that these adult patients usually present mutations of receptor tyrosine kinase (c-KIT), which codes a membrane receptor for stem cell factor, expressed in the surface membrane of mast cells. Mast cells originate in the bone marrow, and they migrate in a precursor state to connective tissue, in which they exert many different functions. Mast cell precursors mature through activation of the receptor CD117, also known as KIT [11]. Although it is accepted that there is a limited KIT mutation spectrum in pediatric mastocytosis, it is still a clonal disease. The c-KIT mutation is seen among 90% of patients with adult mastocytosis. In fact, it has been reported that the rate of c-KIT mutation in pediatric patients can be found in varying ratios, from 0% to 83% [12]. The observation of increased chromosomal breaks in patients with mastocytosis where proliferation may be enhanced is consistent with the current hypothesis that dysmyelopoiesis is related to the c-KIT mutation. This suggests that the relationship between c-KIT–mediated signal transduction and the occurrence of chromosomal abnormalities is perhaps due to a defective repair mechanism [10,13]. Some authors have determined c-KIT mutations in 43% of children with CM [14]. Sporadic mutations at codons 816 and 820, and also several inactivating mutations at codon 839, have been reported in some cases of CM in children [15,16].

The induction of melanocytes determines hyperpigmentation. Pruritus is associated with increased levels of interleukin-31 (IL-31) [17]. Additionally, IL-6 is a marker of M severity [18]. Sometimes, patients with M also develop food allergies: Studies have demonstrated increased expression of TRAF4 gene (a member of the tumor necrosis factor (TNF) receptor-associated factor family, a family of scaffold proteins). Furthermore, allergies to insect venom are associated with the expression of B3GAT1 gene (that encodes the enzyme 3-beta-glucuronosyltransferase 1, and its enzymatic activity creates the CD57 epitope on other cell surface proteins). An important fact is that sometimes (25% of pediatric patients with UP), CM in children is associated with D816V [19,20,21]. The mechanism of action is based on the activation of tyrosine kinase, which leads to cell proliferation [20].

It is known that mast cell hyperplasia may be found in both children with M as well as other hematologic diseases. In M, core bone marrow biopsies (cylindrical sample) evidence focal areas of hyperplasia, eosinophils, mast cells, and early myeloid cells. Moreover, it seems that bone marrow lesions are different in pediatric patients than in adults: Mast cells are small, perivascular, with oval and round nuclei. Other authors have evidenced that mast cell distribution is also essential: Mast cell aggregates are found around blood vessels and throughout the dermis in children [9].

The role of histamine is well known. In healthy children, its plasma levels are 0.3–1.0 ng/mL. Histamine levels increase in diffuse cutaneous mastocytosis (DCM), and in UP, escalates seven times the normal value [22]. However, an absolute correlation has not been found between histamine levels and mast cell load in skin lesions. Another study demonstrated that pediatric patients with high histamine levels (plasma and urine) present more severe bone involvement and change in basal gastric acid concentration [18].

Another enzyme released from mast cells is tryptase (normal levels < 11.5 ng/mL) [23]. Some authors have shown that tryptase levels are higher in DCM than in UP [23]. Serum tryptase levels in DCM patients are high during young infancy (the first two months of life) but tend to decrease toward ages 9–12 months, when the lowest levels are observed [24].

Another argument for this enzyme’s importance is the correlation between the scoring index of M (SCORMA—an index for the management and follow-up) and tryptase levels. Studies support using tryptase for diagnosis and follow-up of CM [24].

### 2.3. Clinical Appearance of CM in Childhood

We describe in the following the most common form of CM in children.

UP is characterized by maculopapular/plaque/nodule/bulla, relatively well-delimited, of different sizes (1–2 cm), the color varying from brown to yellow, located on the scalp, face, trunk, and extremities [16,25]. Usually, these lesions are itchy [26]. Trigger factors, such as cold water, hot baths, or exercise, may cause the lesions to blush [27]. Additionally, pediatric patients sometimes associate with an atopic pathology [28,29]. 

Mastocytomas occur less often than UP in children (10–35%) and present similar to UP, single or multiple brown nodules, but larger. Sometimes, these lesions may vesiculate and blister. Special attention must be paid, as mastocytomas may lead to arterial hypotension or flushing [30,31]. In most cases, no systemic involvement is present [16].

Diffuse cutaneous mastocytosis (DCM) is rare in pediatric patients (1–3% of forms of CM). Lesions are subcutaneous nodules or diffuse bullae, yellow-orange, and may involve the whole skin. In time, the skin may become leathery and thickened, and also hyperpigmentation and dermographism persist. DCM sometimes associates with systemic symptoms, such as anemia, diarrhea, intestinal bleeding, arterial hypotension, and hypovolemic shock. These manifestations are linked to the increased number of mast cell mediators released and absorbed locally and systemically. Some authors have reported lymphadenopathy and hepatomegaly [16,32].

Telangiectasia macularis eruptiva perstants (TMEP) manifests itself exceptionally in children. The eruption might be associated with UP and consists of red, telangiectatic macules in a brown background. However, little data exist about this disease, and further studies are necessary [16,33].

It is important to mention that pediatric patients with CM may develop mast cell mediator-related symptoms. These symptoms appear due to local systemic actions of mast cell mediators. There is no direct correlation between the gravity of the manifestations and the severity of the skin damage. Common reactions include flushing; as for rare symptoms, the following are mentioned: Arterial hypotension, acute episodes of cyanosis and respiratory arrest, and anaphylactic reactions in patients with UP.

Moreover, children with CM may present gastrointestinal symptoms: Diarrhea or abdominal pain. Peptic ulcers or hyperacidity are also reported.

### 2.4. Evaluation of Pediatric Patients with CM

First, the eruption’s clinical examination is critical because it may decide whether the lesions could be part of CM. Thus, it can be one of the four forms of CM: Maculopapular cutaneous mastocytosis (MPCM-UP), DCM, TMEP, or mastocytoma. Always ask the parents/children about systemic symptoms, such as flushing, pruritus, abdominal pain, or diarrhea. Furthermore, complete laboratory examinations are required: Complete blood count, biochemistries, liver function, and, very helpful for the diagnosis, total serum tryptase [9]. 

Sometimes, a lesion’s clinical aspect is not exact, so that a skin biopsy is needed. If the pathological exam does not certainly establish CM, another alternative diagnosis should be considered. Correlating with laboratory tests is mandatory whenever the biopsy findings are typical of CM. Normal values sustain the diagnosis, implying clinical follow-up and basic laboratory tests every 6–12 months. Abnormal tests or lymphadenopathy or hepatosplenomegaly at the physical exam suggest SM. The child should be further evaluated with abdominal ultrasound or computed tomography (for hepatosplenomegaly, lymphadenopathy) and bone marrow biopsy [9].

A specific mark in CM is the Darier sign: If a lesion is stroked, it urticates and rapidly becomes erythematous–edematous and pruritic. This phenomenon is due to physical stimulation, which provides mast cell degranulation [34]. 

Tryptase is a serum marker that can help distinguish between SM and CM, as it is usually normal in CM. However, cases of CM with high levels of this enzyme are also described. The blood should be drawn in a baseline state, and normal levels are 1–11.4 ng/mL. This stands as a minor criterion for the diagnosis of SM. 

Other lab tests are typically regular in CM (only mild eosinophilia is sometimes associated) [32,35,36].

Whenever a skin biopsy is needed, a small punch (3 mm) should be used. The patient should have stopped taking H1 antihistamine. The pathological findings are: Perivascular infiltrates (papillary and upper dermis), interstitial and nodular infiltrates, and sheet-like penetrates in the papillary body and upper reticular dermis. In UP, mast cells have irregular shapes, bilobed nuclei of infiltrating eosinophils. It is important to note that cutaneous biopsy does not bring information about systemic impairment. Additional immunohistochemical tests for tryptase and KIT are of use [31].

### 2.5. Diagnostic Criteria for CM in Childhood

CM is established based on the typical clinical aspect (one of the four described before: UP, mastocytomas, DCM, or TMEP) and skin biopsy with a pathological examination, revealing mast cell infiltrates. Moreover, systemic involvement of the disease should be excluded [9].

An algorithm for the diagnosis of CM in children is reviewed in Table 1.

### 2.6. Differential Diagnoses of CM in Childhood

Depending on the type of skin lesions associated with CM in children, the differential diagnosis involves several dermatological pathologies that are detailed in Table 2 [37,38,39,40,41,42,43,44,45,46,47,48].

### 2.7. Management of CM

Carefulness is advisable in pediatric patients with extensive skin involvement or increased tryptase levels. They may have a lower threshold to anaphylactoid reactions. Self-injectable epinephrine should be limited to cases with high baseline Se-tryptase levels, a history of anaphylactic events, and/or extensive skin involvement or signs of systemic mastocytosis [49,50].

We should always inform the parents/patients about trigger factors: Heat, cold (sudden changes of temperature), humidity, rubbing or pressure of skin lesions, scalp trauma (lesions on the scalp), sleep deprivation, exercise, emotional stress, spicy food, and infectious diseases with fever. Plus, parents should know the medications that can cause mast cell activation: Morphine, codeine, vancomycin, aspirin, ketorolac, and muscle relaxants [51,52,53].

In UP, oral H1 antihistamines are recommended because of their anti-itching and anti-flushing effects. Additionally, H2 antihistamines may help, especially with gastrointestinal symptoms: Abdominal pain, cramping, and diarrhea [54]. H1 antihistamines include diphenhydramine, hydroxyzine, and cetirizine; the child may present adverse effects, such as cardiotoxicity [55]. H2 antihistamines (famotidine and ranitidine) control gastric hypersecretion and peptic ulcer disease. If success lacks, therapy can include proton pump inhibitors [56,57]. 

In selected cases, we can opt for oral cromolyn sodium to control gastrointestinal symptoms. Studies have shown decreased pruritus in several children [9]. Because of frequent side effects, this drug should be introduced with cautious up-dosing [10].

Severe CM forms, such as diffuse bullous disease, or life-threatening forms can benefit from oral methoxypsoralen therapy with long-wave psoralen plus ultraviolet A radiation (PUVA) [58].

In limited CM, a potent dermatocorticoid may have beneficial effects. However, we must pay attention to corticosteroids’ side effects, especially in children: Skin atrophy and adrenocortical suppression, which may develop if a large amount of corticosteroid is applied. Moreover, calcineurin inhibitors, pimecrolimus or tacrolimus, decrease the density of murine cutaneous mast cells and reduce histamine production by inducing mast cell apoptosis. No topical or systemic complications have been observed. As for systemic corticosteroids, only a few studies have proven their efficiency in severe skin disease [57]. We must also note that this drug’s mechanism of action, when it comes to mastocytosis, is redistribution rather than mast cells’ death [59].

Studies have shown that therapy with anti-Ig E, omalizumab, reduces acute episodes in pediatric patients with CM [58].

Prior to a surgical procedure, we recommend reducing anxiety or even preoperative sedation (oral diazepam). To reduce the number of pharmacologic agents, intradermal skin testing of drugs used in anesthesia and relaxing agent (low risk of histamine release) should be used. Moreover, on operation day, guidelines suggest administration of prednisolone intravenous (i.v,) bolus 2 mg/kg (prior to the procedure 1 mg/kg) and clemastine 3 dd 0.05 mg⁄kg orally. In the peri-operative period, it is important to keep adrenaline at hand, as well as isofluran. Postoperatively, with caution, we can opt for acetaminophen or paracetamol [9]. Follow-up of a child with CM can be achieved by calculation of the SCORMA Index. This score includes three parts:

- Part A—the extension of cutaneous involvement:
  1. Anterior view:
    ∘ Head: 4.5
    ∘ Trunk: 18
    ∘ Arms and forearms: 4.5 each
    ∘ Hands: 1 each
    ∘ Genital organs: 1
    ∘ Lower limbs: 9 each
  2. Posterior view:
    ∘ Head: 4.5
    ∘ Trunk: 18
    ∘ Upper limbs: 4.5 each
    ∘ Lower limbs: 4.5 each
- Part B—the activity of lesions, calculated by adding four parameters:

Parameters are marked as 0 = absent; 1 = mild; 2 = moderate; 3 = severe.

Parameters are: Pigmentation/erythema; vesiculation; elevation; positive Darier’s sign.

- Part C—for accompanying and subjective symptoms, a visual analog scale is used (range from 0 to 10), filled out by parents if the patient is under five years.

The five subjective symptoms are triggering factors, flushing, diarrhea, pruritus, and localized bone pain.

The SCORMA Index varies between 5.2 and 100 [60].

## 3. Discussions and Conclusions

There are limited data regarding the long-term follow-up of a child with CM. Childhood mastocytosis has a good prognosis: 50–60% of children improve by adolescence. If mastocytosis persists beyond adolescence, 10% of cases progress into systemic involvement with a guarded prognosis. UP, mastocytoma, and cutaneous involvement only have a good prognosis. Solitary mastocytoma resolves spontaneously in the majority of cases [61]. Studies have shown that CM regresses in 80% of pediatric patients [62,63].

All patients should be evaluated for systemic disease, in the presence of the following risk factors: Monomorphic form, the persistence of skin lesions after puberty, late onset of skin lesion (after two years old), abnormal clinical examination (lymphadenopathy and hepatosplenomegaly), abnormal laboratory tests (anemia, leukopenia, leukocytosis, and blast forms) [64]. Differential diagnosis with other pathologies found in children should be made [37,38]. Additionally, when it comes to the management and follow-up of these children, calculation of the SCORMA score is essential. In conclusion, when choosing the right therapy, we should calculate the SCORMA score and follow the recommendations for every case. [60].

To the best of our knowledge, few reviews about CM in childhood exist, and for this reason, further studies are needed. Clinicians should be aware of this possible diagnosis facing a child with polymorphic eruption. Sometimes, a skin biopsy may be necessary. Moreover, a complete clinical evaluation must be undertaken. 

## Figures and Tables

**Table 1:** Algorithm of cutaneous mastocytosis (CM) diagnosis in children.

Indications	Paraclinical Examination	Findings
Suspected CM	Skin biopsy with pathological exam	3 mm punch; Giemsa staining Aggregates of >20 mast cells ± abnormal morphology D816V c-KIT
Urticaria pigmentosa (UP)/telangiectasia macularis eruptiva perstants (TMEP)/other cutaneous clinical forms	Laboratory tests: Serum tryptase	Repeat every 10–12 months
Symptoms: Gastrointestinal, cyanotic spells, flushing, and syncope Cutaneous lesions persist after puberty Systemic involvement suspected	Abdominal ultrasound	Differ according to systemic mast cell mediator-related symptoms or due to systemic involvement
Mast cell mediator-related symptoms Organomegaly Lymphadenopathy Cutaneous lesions persist after puberty Systemic involvement suspected	Bone marrow biopsy	Differ according to systemic mast cell mediator-related symptoms or due to systemic involvement

**Table 2:** Differential diagnosis of CM in children.

Disease	Cutaneous Manifestations	Other Features
Epidermolysis bullosa	Blisters, dystrophic nails, postinflammatory hyperpigmentation, milia, and atrophy	Gene mutations cause the absence of basement membrane components Types: simplex, junctional, dystrophic, and Kindler syndrome
Impetigo bullosa	Small vesicles and flaccid blisters located on intertriginous areas	Etiology: *Staphylococcus aureus*, group II
Neurofibromatosis type 1	Café-au-lait macules and axillary freckling	Skeletal dysplasias, nervous system tumors (frequently neurofibromas), optic nerve tumors, Lisch nodules, learning disabilities, and attention deficit hyperactivity disorder
Histiocytosis X	40% of patients Papules, brown/purplish Rash Purpuric, vesicular, pustular, and papulo-nodular skin lesions	1–3 years old Most common, limited to 1 organ Bone involvement, frequently Diagnosis: Evaluation of involved tissue and clinical context Sometimes, a biopsy of bone lesion
Non-X histiocytosis of childhood	Head and neck Polymorphic clinical eruption, depending on the form	Histopathologic examination Immunohistochemical differentiation
Juvenile xanthogranuloma	Reddish to yellow-brown	Two years old Gradual involution
Congenital melanocytic nevus	Medium-sized, solitary, geographic, and irregular borders	Present at birth/first few months of life
Postinflammatory hyperpigmentation	Acquired hyper melanosis Macules/patches, located in the same areas as the inflammatory process	Cause: Inflammatory disorders and cutaneous injuries Overproduction of melanin Leukotrienes (LT): LT-C4 and LT-D4
Linear immunoglobulin A bullous dermatosis	Vesicles, blisters, and erosions on the skin and mucous membranes	Subepidermal blister Immunofluorescence microscopy: Ig A linear deposits

## Data Availability

All information included in this review is documented by relevant references.
